# First report of root-knot nematode, *Meloidogyne arenaria*, on lavender in Turkey

**DOI:** 10.21307/jofnem-2020-008

**Published:** 2020-03-17

**Authors:** Tevfik Özalp, Gonca Könül, Önder Ayyıldız, Adnan Tülek, Zübeyir Devran

**Affiliations:** 1 Department of Plant Protection, Faculty of Agriculture, University of Akdeniz, Antalya, Turkey; 2 Trakya Agricultural Research Institute, Edirne, Turkey

**Keywords:** *Lavandula angustifolia*, Molecular marker, PCR

## Abstract

Lavender is a medicinal and aromatic plant that is widely grown in Turkey. Gall symptoms were observed on roots of lavender (*Lavandula angustifolia* Mill.) collected from Kırklareli and Edirne provinces. Egg masses were collected from galled roots. DNA isolated from all samples was screened by species-specific primers belonging to the most common species of root-knot nematodes and *M. arenaria* was the only species that was identified in all of the samples analyzed. This is the first report of *M. arenaria* infecting lavender in Turkey.

Root-knot nematodes are the most damaging group of plant-parasitic nematodes (Gill and McSorley, 2011). Since they have a wide host range worldwide, root-knot nematodes cause serious economic losses in plants (Jones et al., 2013). In addition, they can cause more serious damage by forming disease complexes with soil pathogens (Siddiqui et al., 2014; Lobna et al., 2016).

Medicinal and aromatic plants are widely used in pharmacy and perfumery industries. Lavender (*Lavandula* spp.) is mainly grown and used in medicine, perfumes, cosmetics and food (Lis-Balchin, 2002). Many pests and pathogens attack lavenders during growing periods. Root-knot nematodes are the most common and most important nematodes causing damage to lavender. *Meloidoyne incogita* (Kofoid and White, 1912; Chitwood, 1949) was found infecting *L. hybrida* Rev. from Argentina (Gorustovich et al., 1997) and *L. officinalis* Chaix et Vill. from Egypt (Ibrahim and Mokbel, 2009). In another study, *M. luci* Carneiro, Carneiro et al. (2014) was reported on *L. spica* L. (Carneiro et al., 2014). Lavender species, *L. spica* L. was inoculated with *M. arenaria* (Neal, 1892; Chitwood, 1949) and was a suitable host for this root-knot nematode (Moreno et al., 1990). However, there is no report on root-knot nematodes infecting lavender in Turkey.

In 2019, a survey was carried out in the lavender growing areas in Kırklareli and Edirne provinces of Turkey. The roots of lavender plants with symptoms of stunting were observed and examined. Galled roots were collected from seedlings and examined with a stereo-binocular microscope. The galls were symptoms caused by root-knot nematodes (Fig. 1). Egg masses were separately collected from roots of *L. angustifolia* cultivars, Raya, Hemus, Sevtopolis, Hebar and Yubileina using a small needle. For molecular identification, DNA was extracted from egg masses using the High Pure PCR Template Preparation Kit (Roche). Then, DNA samples were screened by species-specific primers belonging to the most common species of root-knot nematodes. PCR amplifications were carried out using inc-K14F/inc-K14R primers (Randig et al., 2002) and MincF1/MincR1 (Devran et al., 2018) for *M. incognita*; Fjav/Rjav (Zijlstra et al., 2000) for *M. javanica* (Treub, 1885; Chitwood, 1949); Far/Rar (Zijlstra et al., 2000) for *M. arenaria*; JMV1/JMV2/JMVhapla (Wishart et al., 2002) for *M. hapla* (Chitwood, 1949), *M. fallax* Karssen 1996, and *M. chitwoodi* (Golden et al., 1980).

**Figure 1:**
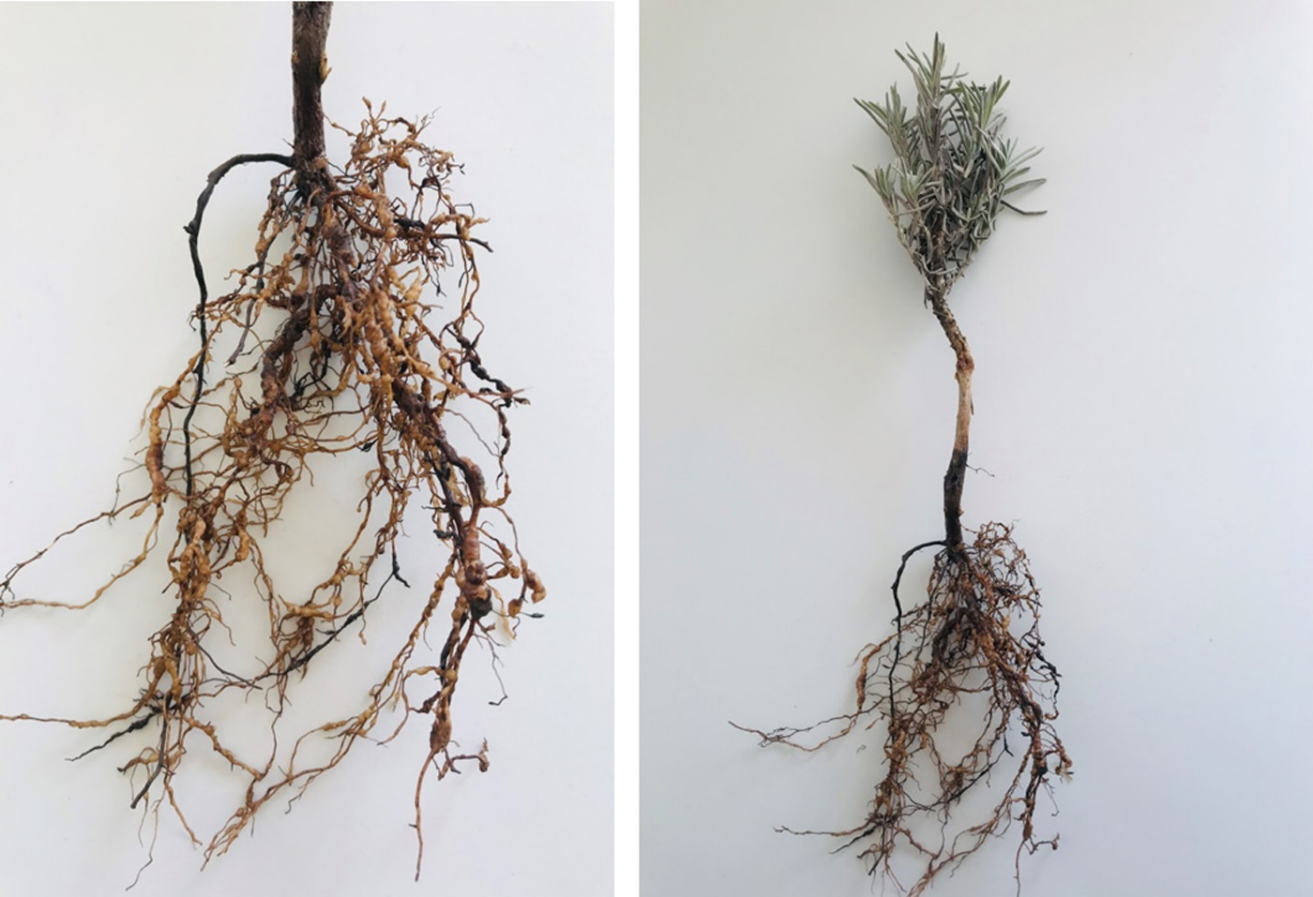
Root-knot nematode galls caused by *Meloidogyne arenaria* (Neal, 1892; Chitwood, 1949) on lavender (*Lavandula angustifolia* Mill.).

PCR was performed in a total volume of 25 μL containing the following: 2.5 μL 10X PCR buffer, 2 mM MgCl2, 200 μM dNTPs, 0.4 μM of each primer, 1 U Taq DNA Polymerase (ABM), 20 ng of DNA and molecular grade water. PCR was performed in a SimpliAmp™ Thermal Cycler (Applied Biosystems, CA, USA) using reaction conditions detailed in cited publications. PCR products were electrophoresed on a 1.5% agarose gel in 1X TAE buffer. Agarose gels were stained with ethidium bromide (0.5 μg/μL) and visualized using the Gel iX Imager (Intas Science, Göttingen, Germany).

In the present study, a total of 10 DNA samples were screened with 5 different primer sets. *M. arenaria*-specific Far/Rar primers only produced expected approximately 420 bp PCR product all samples but other primer sets did not yield in sampled analyzed (Fig. 2). This is the first report of root-knot nematode infecting lavender in Turkey.

**Figure 2:**
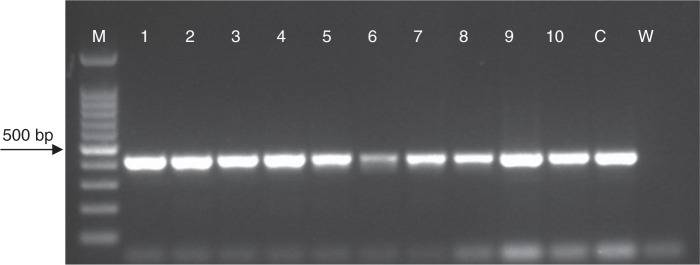
PCR products amplified using primers Far/Rar. M: marker (100 bp DNA Ladder HibriGen), 1–4: samples collected from Kırklareli, 5–10: samples collected from Edirne, C: positive control, W: water.

In conclusion, the present study is the first to report information on the identification root-knot nematodes in lavender crops in Turkey using molecular markers. The results showed that lavender growing fields in Kırıklareli and Edirne provinces were infested with *M. arenaria*. These findings could be used to control root-knot nematodes by screening for resistant cultivars or utilizing crop rotations with non-host plants.
